# An atypical presentation of NSCLC in a nonsmoker: Shoulder pain and upper extremity DVT: A case report

**DOI:** 10.1097/MD.0000000000045965

**Published:** 2025-11-07

**Authors:** Erdoğan Özdemir, Turgay Yilmaz, Pinar Özdemir, Deccane Düzenci, Erman Özdemir

**Affiliations:** a Department of Internal Medicine, Elaziğ Fethi Sekin City Hospital, Elaziğ, Turkey; b Department of Nephrology, Dr. Lütfi Kirdar City Hospital, Istanbul, Turkey; c Department of Intensive Care, Elaziğ Fethi Sekin City Hospital, Elaziğ, Turkey; d Department of Nephrology, Pendik State Hospital, Istanbul, Turkey.

**Keywords:** adenocarcinoma, EGFR mutation, lung neoplasms, venous thrombosis

## Abstract

**Rationale:**

Lung cancer is a leading cause of cancer-related mortality and is often diagnosed late due to nonspecific or extrapulmonary symptoms. Atypical presentations in never-smokers with adenocarcinoma may delay diagnosis. This case highlights the diagnostic value of shoulder pain caused by unprovoked upper extremity deep vein thrombosis (UEDVT) as an early manifestation of thoracic malignancy.

**Patient concerns:**

A 62-year-old male nonsmoker presented with a month-long history of progressive right shoulder pain radiating to the neck, without respiratory symptoms or trauma. The pain persisted despite analgesics.

**Diagnoses:**

Doppler ultrasonography showed acute thrombosis of the right subclavian, axillary, brachial, and internal jugular veins, explaining the shoulder pain. Contrast-enhanced computed tomography (CT) revealed a right hilar mass compressing vascular structures, and positron emission tomography–CT demonstrated mild hypermetabolism (maximum standardized uptake value = 3.1) without metastasis. Endobronchial biopsy confirmed epidermal growth factor receptor–mutant invasive adenocarcinoma.

**Interventions:**

The patient was hospitalized for extensive UEDVT and treated with low-molecular-weight heparin, later switched to the direct oral anticoagulant. No thrombectomy or thrombolysis was required. A multidisciplinary tumor board planned surgical or chemoradiotherapeutic management based on resectability.

**Outcomes:**

Symptoms improved with anticoagulation, and the patient remained clinically stable pending oncologic therapy.

**Lessons:**

Lung adenocarcinoma may rarely present with isolated shoulder pain as the initial symptom. When UEDVT is identified without an evident provoking factor, underlying malignancy should be thoroughly investigated. Additionally, epidermal growth factor receptor–mutant adenocarcinomas may demonstrate only mild fluorodeoxyglucose uptake on positron emission tomography–CT, emphasizing that low metabolic activity does not exclude cancer.

## 1. Introduction

Lung cancer, particularly non-small cell lung carcinoma (NSCLC), typically presents with respiratory symptoms such as chronic cough, hemoptysis, dyspnea, or chest pain. However, a significant proportion of patients may initially exhibit nonspecific or extrapulmonary manifestations, which often contribute to diagnostic delays and worse clinical outcomes.^[1]^ Atypical presentations, including musculoskeletal pain, neurological symptoms, or thromboembolic events, are more frequently reported in adenocarcinomas and in patients without traditional risk factors such as smoking.^[2,3]^

Upper extremity deep vein thrombosis (UEDVT) is an uncommon but increasingly recognized paraneoplastic phenomenon, accounting for <10% of all deep vein thrombosis (DVT) cases.^[4]^ In the absence of catheterization, trauma, or known thrombophilic disorders, idiopathic UEDVT should raise clinical suspicion for occult malignancy.^[5]^ Several studies have reported thoracic tumors—particularly lung adenocarcinomas—as underlying causes of unprovoked UEDVT due to local vascular compression and prothrombotic tumor-mediated mechanisms.^[6,7]^ Herein, we present a diagnostically challenging case of NSCLC in a never-smoker male, initially presenting with isolated shoulder pain and extensive UEDVT.

## 2. Case presentation

A 62-year-old retired male schoolteacher presented to the internal medicine outpatient clinic with a 1-month history of progressive right shoulder pain radiating to the lateral neck. The pain was described as dull, non-positional, and persistent throughout the day and night, with no relief from rest or activity modification. He denied any history of trauma, overuse, or recent physical exertion. One month earlier, he had been evaluated at a physical therapy and rehabilitation center, where he was provisionally diagnosed with musculoskeletal strain and prescribed nonsteroidal anti-inflammatory drugs and muscle relaxants. Despite this treatment, his symptoms steadily worsened, prompting further evaluation.

On review of systems, the patient denied cough, dyspnea, hemoptysis, chest pain, fever, night sweats, or weight loss. He had no past medical history of hypertension, diabetes, vascular disease, or cancer. He reported no regular medication use, had never smoked, and consumed alcohol only occasionally. There was no known family history of malignancy or thrombotic disorders.

On physical examination, the patient was hemodynamically stable and afebrile. Localized tenderness was noted over the right supraclavicular and infraclavicular regions, and a palpable, mobile, tender nodular mass approximately 1 cm in diameter was identified along the anatomical course of the subclavian vein. There was no erythema, warmth, or skin discoloration. Neurological examination of the right upper extremity revealed normal muscle strength, tone, and reflexes, with no sensory deficits. Cardiopulmonary and abdominal examinations were unremarkable.

Initial laboratory investigations demonstrated a mild elevation in inflammatory markers, with erythrocyte sedimentation rate of 38 mm/h and C-reactive protein of 14 mg/L. Complete blood count, renal and hepatic function tests, coagulation parameters, and d-dimer levels were within normal limits. Given the localized swelling, a venous Doppler ultrasonography of the upper extremity was performed. It revealed acute, occlusive thrombosis involving the right subclavian, axillary, brachial, and internal jugular veins. No collateral venous flow was detected.

The patient was promptly admitted to the internal medicine service for management of extensive UEDVT. Therapeutic anticoagulation was initiated with enoxaparin at a dose of 1 mg/kg subcutaneously every 12 hours. The patient was monitored for bleeding risk and platelet counts throughout hospitalization. Vascular surgery consultation ruled out the need for emergent thrombectomy or catheter-directed thrombolysis, given the absence of limb-threatening ischemia or phlegmasia.

Given the extent of thrombosis and absence of provoking factors such as trauma, central venous catheterization, malignancy, or thrombophilia, further evaluation was pursued. A comprehensive thrombophilia panel, including protein C and S levels, antithrombin III, Factor V Leiden mutation, prothrombin gene mutation, antiphospholipid antibodies, and homocysteine levels, yielded normal results.

**Figure 1.**
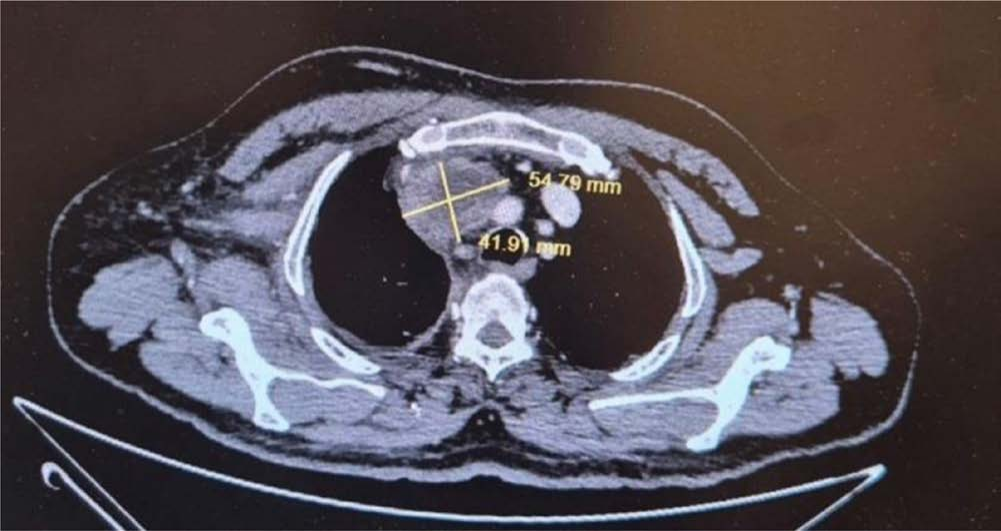
The patient’s contrast-enhanced CT image. Contrast-enhanced thoracic computed tomography (CT) demonstrating a well-defined soft tissue mass measuring approximately 55 × 40 mm in the right hilar region, compressing the adjacent subclavian vein and upper mediastinal vascular structures.

Considering the idiopathic nature of thrombosis, malignancy screening was initiated. Contrast-enhanced thoracic computed tomography (CT) revealed a well-defined, centrally located right hilar mass measuring approximately 55 × 40 mm, which compressed adjacent vascular structures, including the right subclavian vein and superior vena cava (Fig. 1). No obvious lung parenchymal involvement was observed. Subsequent Positron emission tomography–CT confirmed the presence of a hypermetabolic lesion (SUV max: 3.1) corresponding to the same region, with no distant metastasis or lymphadenopathy.

The patient was referred to the pulmonology department. Bronchoscopy with endobronchial ultrasound-guided transbronchial needle aspiration was performed. Histopathological examination confirmed invasive lung adenocarcinoma with an acinar-predominant pattern. Immunohistochemistry revealed strong positivity for TTF-1 and Napsin A, supporting pulmonary origin. Molecular analysis identified a targetable epidermal growth factor receptor (EGFR) exon 19 deletion and PD-L1 expression at 35%, serving as potential biomarkers for future systemic therapy. Although radiologic imaging did not reveal overt parenchymal infiltration, histologic assessment indicated that the tumor originated from the bronchial mucosa with extension into the adjacent lung parenchyma. Following diagnosis, the case was reviewed by a multidisciplinary tumor board. Comprehensive staging confirmed localized disease, classified as T3N0M0 (stage IIB) according to the 8th edition of the TNM classification, without distant metastasis. Depending on mediastinal staging and surgical resectability, curative-intent management would consist of either surgery or concurrent chemoradiotherapy, with molecular findings reserved to guide adjuvant—or, if needed, salvage—systemic therapy.

Given the diagnosis of cancer-associated thrombosis, a long-term anticoagulation strategy was planned. He was transitioned to oral direct anticoagulant therapy (apixaban 5 mg BID) at discharge, with a recommended minimum duration of 6 months in line with American Society of Clinical Oncology guidelines. The decision to switch from low-molecular-weight heparin to a direct oral anticoagulant was based on current evidence and guideline support indicating comparable efficacy and safety, improved patient comfort by avoiding long-term injections, and the patient’s preserved renal function and low bleeding risk profile.^[6]^ Hematology consultation advised reevaluation of anticoagulation continuation based on response to oncologic treatment and bleeding risk.

Following diagnosis and staging, the patient’s symptoms improved under anticoagulant therapy, and no new thrombotic events were observed during hospitalization. Once the diagnostic workup was completed and the disease was confirmed as invasive lung adenocarcinoma, the patient was referred to the oncology department for further management. He remains under regular outpatient follow-up. The clinical course and laboratory findings are summarized in Table 1.

**Table 1:** Clinical and laboratory summary.

Date/interval	Clinical events and interventions
~1 month before presentation	Onset of progressive right shoulder pain
	Radiating to the neck
~3 weeks before presentation	Initial evaluation at the physical therapy center
	Misdiagnosed as a musculoskeletal strain
	Treated with NSAIDs and muscle relaxants
Day 0 (presentation)	Attended internal medicine clinic
	Physical exam revealed tenderness and a palpable 1 cm nodule over the right subclavian vein
Day 0	Doppler ultrasonography: acute, occlusive thrombosis of the right subclavian, axillary, brachial, and internal jugular veins
Hospital admission	Therapeutic anticoagulation initiated
	Enoxaparin 1 mg/kg SC BID
Hospital stay	Thrombophilia panel negative
	No inherited/acquired disorders detected
Day 3–5	Contrast-enhanced thoracic CT: 55 × 40 mm right hilar mass compressing adjacent vascular structures
Shortly after	PET-CT:
	Mildly hypermetabolic lesion (SUVmax 3.1)
	No distant metastasis
Following PET-CT	EBUS-TBNA biopsy:
	Invasive adenocarcinoma (acinar-predominant)
	TTF-1 and Napsin A positive
	EGFR exon 19 deletion; PD-L1 35%
Post-diagnosis	Transition to oral apixaban 5 mg BID
	Oncology referral for curative-intent surgery vs. concurrent chemoradiotherapy
Post-discharge follow-up	Clinical improvement, resolution of pain and swelling
	Apixaban well-tolerated without bleeding
	The oncology tumor board confirmed localized disease
	Curative-intent management planned (surgery if operable, chemoradiotherapy if unresectable)

CT = computed tomography, DOAC = direct oral anticoagulant, EBUS-TBNA = endobronchial ultrasound-guided transbronchial needle aspiration, EGFR = epidermal growth factor receptor, LMWH = low-molecular-weight heparin, NSAIDs = nonsteroidal anti-inflammatory drugs, PET–CT = Positron emission tomography–computed tomography, SC BID = subcutaneous, twice daily, TTF-1 = thyroid transcription factor-1.

## 3. Discussion

Lung cancer continues to represent a major global health burden and remains the leading cause of cancer-related mortality, accounting for approximately 1.8 million deaths annually worldwide.^[8]^ While smoking is the most established risk factor, an increasing proportion of lung cancer cases—particularly adenocarcinomas—are being diagnosed in never-smokers, especially among older adults and females.^[9]^ The presented case illustrates a diagnostically challenging scenario in which a nonsmoking elderly male developed nonspecific symptoms unrelated to the respiratory system, ultimately found to be due to NSCLC.

Classically, lung cancer manifests with respiratory and constitutional symptoms such as chronic cough, hemoptysis, unexplained weight loss, dyspnea, chest pain, or hoarseness. The presence of risk factors such as tobacco exposure or a positive family history usually prompts timely radiologic investigation.^[3]^ However, when the initial symptoms are atypical or extra-thoracic—such as isolated musculoskeletal pain—the diagnostic process may be significantly delayed, as was the case in our patient.

In this case, the patient is presented with progressive right shoulder and neck pain without respiratory complaints or systemic symptoms. Initial management targeted a presumed musculoskeletal etiology, reflecting a common pitfall in routine clinical practice. Persistent symptoms and the emergence of a localized venous swelling eventually prompted further imaging, which revealed extensive UEDVT and subsequently a mediastinal mass consistent with NSCLC.

Venous thromboembolism, including deep vein thrombosis and pulmonary embolism, is a well-recognized paraneoplastic phenomenon and may be the first manifestation of occult malignancy. The pathophysiology is multifactorial, involving tumor-induced activation of coagulation pathways, endothelial injury, and cytokine-mediated inflammation.^[4]^ While lower extremity DVT is more common, UEDVT constitutes approximately 10% of all DVT cases and is frequently associated with central venous catheters or thoracic malignancies.^[5]^

Unprovoked UEDVT, particularly in the absence of central venous catheterization or thrombophilia, should raise suspicion for occult malignancy. Current guidelines, including the 2024 National Comprehensive Cancer Network Clinical Practice Guidelines in Oncology for Venous Thromboembolic Disease, recommend malignancy screening in patients with unprovoked venous thromboembolism, as cancer is identified in approximately 10% of such cases within 1 year of diagnosis.^[10]^ Similarly, the American Society of Clinical Oncology Clinical Practice Guideline on Cancer-Associated Thrombosis emphasizes that idiopathic UEDVT warrants a thorough evaluation for underlying malignancy, including thoracic imaging in high-risk populations.^[6]^ In our case, the absence of conventional risk factors for thrombosis—including catheterization, recent surgery, or thrombophilia—prompted a malignancy workup that led to the diagnosis. These recommendations, as the patient’s UEDVT led to the diagnosis of NSCLC, underscore the critical role of guideline-adherent evaluation in unexplained thrombosis. The recommended guidelines mentioned have been specifically summarized for the case in Table 2.

**Table 2:** Guideline recommendations for malignancy screening in UEDVT.

Guideline	Recommendation	Relevance to this case
NCCN (2024)	“In unprovoked VTE (particularly upper extremity DVT), malignancy screening should be considered.”	The patient presented with UEDVT without identifiable risk factors; thoracic CT revealed NSCLC.
ASCO (2023)	“Idiopathic UEDVT warrants evaluation for occult malignancy, including thoracic imaging in high-risk populations.”	PET-CT and EBUS-TBNA confirmed a hilar mass, consistent with NSCLC.

ASCO = American Society of Clinical Oncology, CT = computed tomography, EBUS-TBNA = endobronchial ultrasound-guided transbronchial needle aspiration, NCCN = National Comprehensive Cancer Network, NSCLC = non-small cell lung cancer, PET–CT = Positron emission tomography–computed tomography, UEDVT = upper extremity deep vein thrombosis, VTE = venous thromboembolism.

Importantly, the anatomical proximity of the right hilar mass to the subclavian and jugular veins in this patient supports a dual mechanism: direct vascular compression and local hypercoagulability, which contribute to thrombus formation. The mass effect and venous stasis likely played a synergistic role. Similar cases have been described in the literature, albeit rarely. In one such report by Díaz-Abad et al, shoulder pain was the only presenting symptom of a right upper lobe lung cancer, which had invaded the brachial plexus and subclavian vessels.^[7]^

In EGFR-mutant lung adenocarcinomas, as in our case, fluorodeoxyglucose uptake on Positron emission tomography–CT may be deceptively low, and SUVmax values may not accurately reflect the true malignant potential. The modest SUVmax (3.1) observed in our patient with an EGFR exon 19 deletion is consistent with a 2021 meta-analysis of 15 studies including 3574 patients, which demonstrated the limited diagnostic utility of SUVmax in predicting EGFR mutation status (sensitivity 70%, specificity 59%, diagnostic odds ratio 3.5).^[11]^

These results highlight a well-recognized limitation: EGFR-mutant tumors often exhibit reduced metabolic activity on PET imaging. Therefore, in never-smokers or patients with atypical presentations and low fluorodeoxyglucose uptake, malignancy should not be excluded based solely on PET findings. Histopathological and molecular confirmation remains essential, and early tissue sampling—via endobronchial ultrasound-guided transbronchial needle aspiration or CT-guided biopsy—should be strongly considered to ensure timely and appropriate treatment planning.

In Turkey, lung cancer remains one of the leading causes of cancer-related mortality, with adenocarcinoma being the predominant histological subtype among nonsmokers. According to the Turkish Cancer Statistics 2022 report and GLOBOCAN 2020 estimates, approximately 17% to 20% of lung cancer cases occur in never-smokers, with a higher incidence noted in elderly and female populations.^[12,13]^ These epidemiological trends reinforce the need for heightened clinical vigilance and early imaging in patients without traditional risk factors.

This case highlights several critical teaching points. First, persistent musculoskeletal symptoms that are refractory to standard therapy, particularly in older adults, warrant a broader differential diagnosis and appropriate imaging. Second, the presence of unprovoked or extensive venous thrombosis—especially in unusual locations like the upper extremity—should raise suspicion for an underlying malignancy even in the absence of traditional cancer risk factors. Third, early utilization of thoracic imaging, including contrast-enhanced CT, can be lifesaving in uncovering otherwise silent thoracic malignancies.

Moreover, this case serves as a reminder that nonsmoking status does not preclude the possibility of lung cancer. Environmental exposures (e.g., radon, air pollution), genetic susceptibility, and other unknown factors may contribute to oncogenesis in never-smokers.^[2]^ Given the rising incidence of lung cancer in this population, particularly in Asia and Eastern Europe, clinicians must maintain a high index of suspicion in patients with unexplained symptoms and no obvious risk profile.

Timely diagnosis is essential, as the stage at diagnosis remains the most important prognostic factor in NSCLC. Early-stage disease (I–IIIA) is amenable to curative-intent treatment, whereas delays may lead to advanced-stage presentation and limited therapeutic options.^[14]^ In this case, malignancy was detected at a potentially treatable stage, underscoring the value of astute clinical reasoning and early imaging in atypical presentations.

## 4. Strengths and limitations

This case highlights an uncommon presentation of non-small cell lung cancer in a never-smoker, emphasizing the diagnostic relevance of unprovoked upper extremity deep vein thrombosis and persistent musculoskeletal pain. The report integrates radiologic, pathologic, and molecular findings, providing translational insight into EGFR-mutant disease.

However, several limitations should be acknowledged. As a single-patient case report, the findings cannot be generalized. The follow-up duration was limited, precluding long-term outcome assessment. Additionally, histopathological images could not be included because digital image records were unavailable. Future multi-patient studies are warranted to validate these observations and further clarify the link between thoracic malignancy and idiopathic upper extremity thrombosis.

## Author contributions

**Conceptualization:** Erdoğan Özdemir.

**Data curation:** Erdoğan Özdemir, Turgay Yilmaz, Deccane Düzenci.

**Formal analysis:** Erdoğan Özdemir, Pinar Özdemir.

**Investigation:** Erman Özdemir.

**Methodology:** Deccane Düzenci.

**Project administration:** Erdoğan Özdemir, Erman Özdemir.

**Resources:** Erdoğan Özdemir, Deccane Düzenci.

**Supervision:** Erman Özdemir.

**Validation:** Erdoğan Özdemir, Turgay Yilmaz, Pinar Özdemir.

**Visualization:** Erdoğan Özdemir.

**Writing – original draft:** Erdoğan Özdemir.

**Writing – review & editing:** Erdoğan Özdemir, Erman Özdemir.
